# Pembrolizumab-Induced Lichen Planus in a Patient With Non-Small-Cell Lung Carcinoma (NSCLC) That Correlates to Therapeutic Response

**DOI:** 10.7759/cureus.31454

**Published:** 2022-11-13

**Authors:** Aasim Sehbai, Muhammad A Hamid, Zainab Ibrahim

**Affiliations:** 1 Hematology and Oncology, Alabama Cancer Care (ALCC), Anniston, USA; 2 Internal Medicine, University of South Dakota Sanford School of Medicine, Sioux Falls, USA; 3 Neurosciences, University of Alabama at Birmingham, Birmingham, USA

**Keywords:** check point inhibitors, immunotherapy, non-small cell lung cancer, lichen planus, pembrolizumab

## Abstract

Immune checkpoint inhibitors are increasingly being used in the treatment of various solid organ and hematologic malignancies. Dermatologic toxicities associated with programmed cell death protein-1 (PD-1) and programmed death ligand-1 (PD-L1) therapy have been widely reported in the literature. Lichen planus is an inflammatory disease frequently seen in areas of the skin and oral mucous membrane lining. This autoimmune disorder is T-cell mediated with multiple contributing factors like emotional stress, genetic predisposition, isotopic response, or drugs. With increasing use of immunotherapy, early recognition and prompt treatment of associated adverse events are critical to ensure patient safety. Cutaneous toxicities are among the most commonly observed adverse events with this class of drugs. Here, we report a case of lichen planus in a 66-year-old male patient receiving pembrolizumab for stage IV non-small cell lung cancer (NSCLC). He was diagnosed 56 months ago with advanced lung cancer with brain metastasis. He has received 62 cycles of pembrolizumab and continues to be in complete clinical and radiographic remission. Pembrolizumab is a drug that helps immune cells in killing cancer cells by binding to the PD-1 protein. This case highlights the potential cutaneous side effects that may result in a patient with pembrolizumab and the fact that it can serve as a "clinical biomarker" and show therapeutic effectiveness of the treatment.

## Introduction

Pembrolizumab (also called Keytruda), is an immunotherapy medication approved for various cancers. FDA has approved it for 18 different cancer indications. Although the exact mechanism for this treatment is unknown, it is thought to work by blocking the programmed cell death protein-1 (PD-1) on the surface of immune T-cells, triggering the body’s immune system to kill cancer cells [1]. Anti-PD-1 therapy, in some instances, is followed by adverse dermatological events. One such disease is lichen planus, in which lesions of the skin are formed on the skin or mucosal surfaces [2]. This report describes the presentation of lichen planus on a patient receiving pembrolizumab and its management. We also postulate that lichen planus and lichenoid reactions, although an adverse effect, may indicate that it is a clinical biomarker for activity of the drug and may lead to long, durable and sustained therapeutic responses and better survival for patients. 

## Case presentation

**Figure 1:**
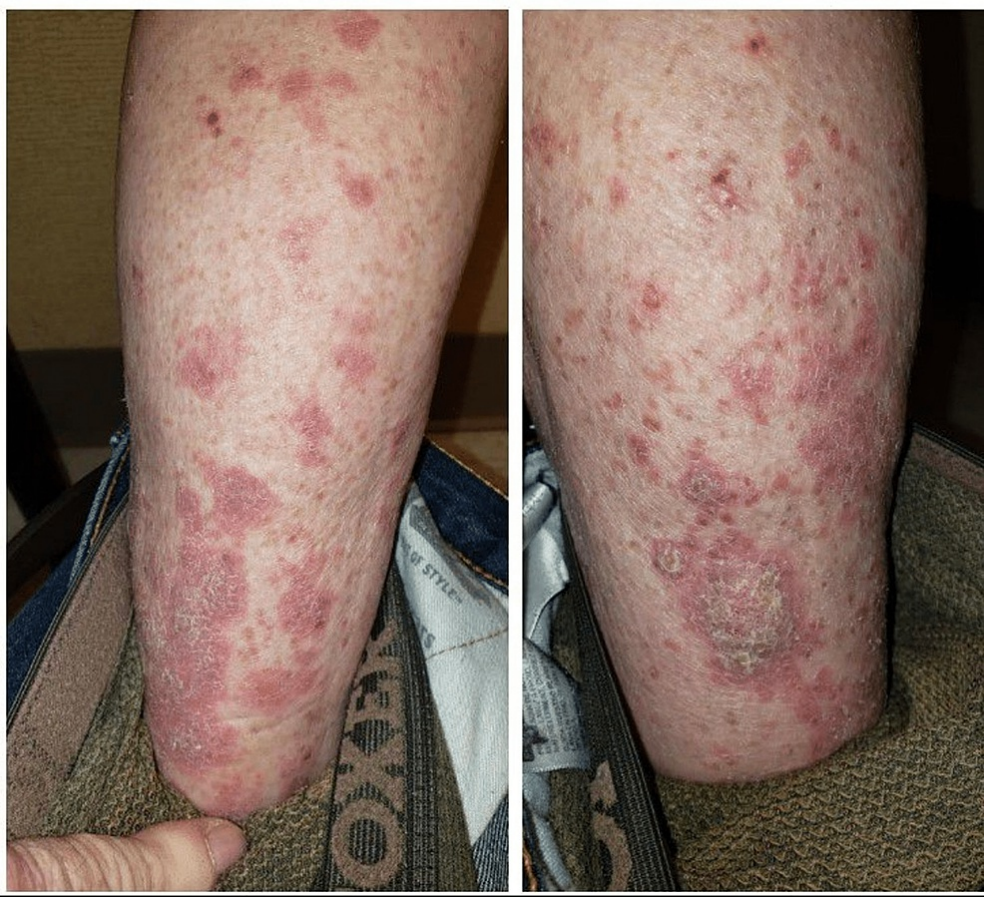
Lichen planus seen on lower extremities with use of pembrolizumab

The patient was 66 years old when he presented with shortness of breath, hemoptysis, dysphagia and a 40-pound weight loss. He had smoked for 40 years (60-pack years). Chest X-ray showed a mass in the right lower lobe (RLL) and a CT Chest confirmed that it was a 6.3 x 5.7 x 5.9 cm mass in RLL. There was mediastinal lymphadenopathy and presence of bilateral adrenal metastasis. He also had bulky neck adenopathy and early symptoms of superior vena cava (SVC) syndrome. Fine needle aspiration (FNA) from the neck lymph node confirmed that he had a poorly differentiated lung adenocarcinoma. Programmed death ligand-1 (PD-L1) expression was 80% positive. He did not have any epidermal growth factor receptor (EGFR), anaplastic lymphoma kinase (ALK) or other actionable mutations on his molecular profile. He did have metastasis in the right temporal lobe of the brain which was very small and not associated with vasogenic edema. Due to SVC syndrome and his compromised respiratory status, decision was made to start systemic chemo-immunotherapy. He received carboplatin, pemetrexed and pembrolizumab combination every three weeks for six cycles. He showed an excellent clinical and radiographic response and his neck adenopathy resolved and a re-staging scan after six cycles showed complete resolution of lung mass, adrenal metastasis as well as brain metastasis. We decided to continue pembrolizumab monotherapy every 21 days. After staying on pembrolizumab for 19 months, he started noticing a rash, itching and eczema in his inner thighs and legs (Figure 1). We treated it with some local steroids and antihistamine drugs with mild improvement. The rash then got worse involving bilateral lower extremities, lower abdomen and was causing pruritis. Punch biopsy done by a dermatologist confirmed that it was lichen planus and was felt to be related to immunotherapy pembrolizumab. It was presenting as red, scaly plaques on his legs and buttocks and severe pruritis. Pembrolizumab was held for 12 weeks which led to an 80% improvement in the rash. Pembrolizumab was carefully re-introduced but currently he is getting it every eight weeks and tolerating it well. Recent PET scan and MRI Brain show he is in complete remission. 

## Discussion

Lichen planus is a mucocutaneous disorder that may affect areas of the skin either simultaneously or sequentially. The pathogenesis of this disease remains unknown, but it appears to be associated with autoimmunity. The basal keratinocyte degeneration seen in lichen planus is a trait of CD8+ T lymphocytes, an important part of the infiltrates present alongside damaged keratinocytes in the epithelium [3]. With the immunotherapy approach in cancer treatment, immune-related adverse effects (IRAEs) have become increasingly frequent. Out of the large variety of IRAEs, cutaneous reactions are most frequently linked to checkpoint inhibitors. Studies show that 8.7% of patients receiving anti-PD-1 therapy with melanoma formed adverse skin reactions. Out of 42% of these patients, pembrolizumab was linked to the patients' adverse skin events [4]. This chronic inflammatory disease appears as white papules, erosions, or blisters. The mechanism by which these outcomes may occur is not known but is thought to be mediated by T cells [5]. In cases when this method of therapy results in severe cutaneous toxicities, current guidelines suggest that patients should hold back on immunotherapy and instead be treated with systemic steroids. If the results do not appear to be life-threatening, there is no need for the permanent discontinuation of immunotherapy, despite there being a possibility for toxicity reoccurring upon the checkpoint inhibitor [6]. Although the pathogenesis of the lichenoid reaction as a cutaneous IRAE remains to be elucidated, several mechanisms have been proposed; for instance, the reaction may be mediated by an unmasked antigen, it may be generated by generalized upregulation of the immune system, and/or a neoantigen may be directly created by the anti-PD-1 antibody [7]. We also postulate that in non-small cell lung cancer, which has strong expression of PD-L1 protein, the appearance of lichen planus or other lichenoid reactions means it is a biomarker for response to immunotherapy and with management of skin toxicity, immunotherapy can be resumed again carefully. 

## Conclusions

In conclusion, pembrolizumab is a very commonly used immunotherapy drug approved in multiple cancers and has revolutionized cancer therapy for both early- and late-stage cancers, as patients are living longer and achieving better survival rates. Immune-mediated side effects of pembrolizumab can be seen like skin toxicity and lichen planus is an uncommon presentation. In our patient, lichen planus was successfully treated by interruption of the drug and then reintroducing it every eight weeks instead of every three weeks. This seems to be an effective strategy that can be utilized in clinical scenarios like ours. 
